# Application of Fresnel Zone Plate Focused Beam to Optimized Sensor Design for Pulse-Echo Harmonic Generation Measurements

**DOI:** 10.3390/s19061373

**Published:** 2019-03-19

**Authors:** Hyunjo Jeong, Hyojeong Shin, Shuzeng Zhang, Xiongbing Li, Sungjong Cho

**Affiliations:** 1 Department of Mechanical Engineering, Wonkwang University, Iksan, Jeonbuk 54538, Korea; hjshin9504@naver.com (H.S.); cho-sungjong@naver.com (S.C.); 2 School of Traffic and Transportation Engineering, Central South University, Changsha 410075, China; zhangshuzeng123@163.com (S.Z.); lixb213@csu.edu.cn (X.L.)

**Keywords:** focused beam, second harmonic generation (SHG), nonlinear parameter, Fresnel zone plate (FZP), pulse-echo method

## Abstract

In nonlinear acoustic measurements involving reflection from the stress-free boundary, the pulse-echo method could not be used because such a boundary is known to destructively change the second harmonic generation (SHG) process. The use of a focusing acoustic beam, however, can improve SHG after reflection from the specimen boundary, and nonlinear pulse-echo methods can be implemented as a practical means of measuring the acoustic nonlinear parameter (*β*) of solid specimens. This paper investigates the optimal sensor design for pulse-echo SHG and *β* measurements using Fresnel zone plate (FZP) focused beams. The conceptual design of a sensor configuration uses separate transmission and reception, where a broadband receiver is located at the center and a four-element FZP transmitter is positioned outside the receiver to create a focused beam at the specified position in a solid sample. Comprehensive simulations are performed for focused beam fields analysis and to determine the optimal sensor design using various combinations of focal length, receiver size and frequency. It is shown that the optimally designed sensors for 1 cm thick aluminum can produce the second harmonic amplitude and the uncorrected nonlinear parameter corresponding to the through-transmission method. The sensitivity of the optimal sensors to the changes in the designed sound velocity is analyzed and compared between the odd- and even-type FZPs.

## 1. Introduction

Nonlinear acoustic effects in metallic materials appear due to the anharmonicity of the crystal lattice and microstructural features such as dislocations and precipitates when strong waves propagate in the interior of the material. More specifically, harmonic waves corresponding to integer multiples of the incident fundamental wave frequency are generated and are called nonlinear acoustic waves. Nonlinear acoustics has received considerable attention in recent decades in view of the importance of ensuring the reliability and integrity of key structural components used in various engineering disciplines. Second harmonic generation (SHG) is most widely measured among these harmonics to assess the acoustic nonlinear parameter, *β*, which is a collective and quantitative measure of microstructural changes and damage state due to fatigue, creep or plastic deformation. The nonlinear parameter of a material is defined by the amplitude ratio of the second harmonic to the square of the fundamental wave. 

The nonlinear parameters of solid media are predominantly measured using the longitudinal wave in the through-transmission (T-T) setup [1,2,3,4], which needs to approach both sides. The T-T technique therefore may have limitations when applied to field measurements. For practical applications, pulse-echo measurements are preferred, which allow the single-side access of test components with stress-free surfaces. The problem with using the second harmonic wave reflected from the stress-free boundary is that such a boundary destructively alters the SHG process and consequently makes it difficult to obtain reliable results for *β*. In the case of a pure plane wave, the second harmonic generated during forward propagation will diminish to zero upon returning to its origin after reflection from the stress-free boundary [5,6]. 

The focused beam is considered an efficient way to optimize the pulse-echo nonlinear wave field with stress-free boundaries because such beams exhibit a significantly different phase behavior from plane waves [7]. According to our recent work, the phase difference between the newly generated and reflected second harmonics was found to be about π/2 after being reflected from the stress-free boundary [8]. Thus, the received second harmonic is greatly improved due to the reinforcing addition of these two components and is measurable in the pulse-echo configuration. Although the study was performed in fluids, where focused transducers are easy to employ, we can also use other methods to focus the beam in solid samples.

In general, it is difficult to use spherical focusing probes on solid specimens with flat surfaces. The typical methods of generating a focused beam in such specimens include the use of phased arrays [9] and Fresnel zone plates (FZPs) [10,11]. Ultrasonic phased arrays consist of an array of a small piezoelectric element and can generate focused sound beams by varying the relative time delays of the driving pulses. FZPs use the diffraction phenomenon for focusing a planar wave into a certain location. FZPs consist of a set of radially symmetrical zones which alternate between opaque and transparent. The zones can be spaced such that the diffracted wave constructively interferes at the desired focus point. Classical FZPs have been widely used in the field of acoustics for focusing water-coupled or air-coupled transducers due to their good focusing performance [12,13,14,15,16,17]. As far as we know, FZP sensors have never been applied to the nonlinear parameter measurement of solid samples with improved SHG in the pulse-echo mode.

The outline of this paper is as follows: Section 2 introduces the design concept for a FZP sensor in which a FZP transmitter of multiple elements and a broadband receiver are separately used for the generation of a focused beam and the reception of signals reflected from the stress-free specimen boundary. The SHG process in the pulse-echo setup is briefly described based on the quasilinear theory. Section 3 presents the quasilinear solutions for the Khokhlov–Zabolotskaya–Kuznetsov (KZK)-type equation that accounts for the combined effects of nonlinearity, diffraction and attenuation in isotropic solids. The beam fields for the single element radiation from the FZP transmitter are expressed in closed forms and the total amplitudes of the received fundamental and second harmonic waves are obtained by summing the contributions from all the FZP elements. The formula for the nonlinear parameter, β, is then defined with the total correction that incorporates the combined effects of reflection, attenuation and diffraction. Section 4 presents the effects of FZP types and FZP zone numbers on the focal properties of FZP transmitters. In Section 5, comprehensive simulations are performed for focused beam field analysis and optimal sensor design using various combinations of focal length, receiver size and frequency. It is shown that the optimally designed sensors for a given sample can produce the desired second harmonic amplitude and the resulting β value corresponding to the through-transmission setup. The sensitivity of the optimal sensors to the changes in the designed sound velocity are compared between the odd- and even-type FZPs. Finally, Section 6 concludes the paper.

## 2. Design Concept of FZP Sensors

The design objective of FZP sensors is that when such sensors are actually fabricated and used for SHG measurement in the pulse-echo mode, the sensor must produce the received second harmonic amplitude and the resulting nonlinear parameter value corresponding to the through-transmission method. The term “SHG measurement” is used here to mean second harmonic generation and the measurement of the uncorrected nonlinear parameter. In order to meet these requirements, the conceptual design of FZP sensors was performed first. In the SHG measurements, the bandwidth of the receiver probe should be wide enough to receive both fundamental and second harmonic components from the output signal. In addition, high voltage excitation is necessary to generate the second harmonic, and it is not easy to select a piezoelectric material that satisfies both the high voltage and wide band characteristics. 

**Figure 1:**
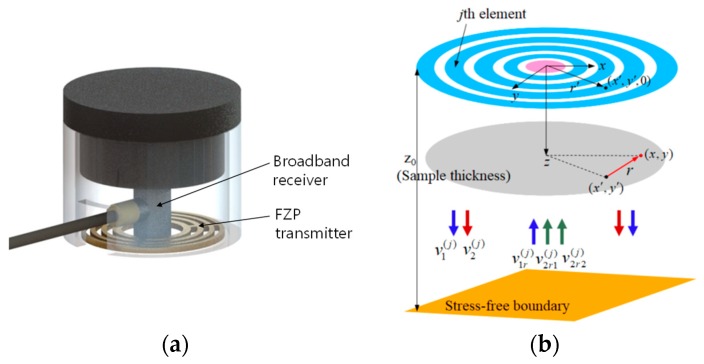
Schematic of a Fresnel zone plate (FZP) sensor and the second harmonic generation (SHG) process in the pulse-echo mode: (**a**) Conceptual design of the FZP sensor and (**b**) radiation from the *j*th element of a FZP transmitter, reflection from the planar stress-free boundary and reception by a circular receiver placed at the center.

One possible solution to this problem is to use a separate transmitter and receiver. That is, a broadband receiver located at the center and a FZP transmitter of multiple elements located outside the receiver to produce a focused beam at the designed position in a solid specimen. The final configuration of the FZP sensor for pulse-echo SHG measurements consisted of an outer FZP transmitter and a central receiver, as shown in Figure 1a. Classical FZPs are either odd plate-type or even plate-type, and multiple elements in the Fresnel zone number are employed in each type.

Figure 1b shows the schematic of the pulse-echo SHG process when the *j*th element of the FZP transmitter radiates a longitudinal (L) wave beam into a solid medium. The FZP transmitter is composed of *N* multiple elements. The SHG process described here is based on the quasilinear theory [18,19] and the total SHG is therefore given by adding the contribution from self-interaction of the fundamental wave in each element. In Figure 1b, $v_{1}^{\left(j\right)}$ is the propagating fundamental wave of frequency *f* due to *j*th element radiation. Here, v denotes the particle velocity. $v_{2}^{\left(j\right)}$ is the generated second harmonic wave due to the forcing of $v_{1}^{\left(j\right)}$. $v_{1r}^{\left(j\right)}$ denotes the reflected fundamental wave when the wave $v_{1}^{\left(j\right)}$ hits the boundary. $v_{2r1}^{\left(j\right)}$ is the reflected second harmonic wave when the wave $v_{2}^{\left(j\right)}$ hits the boundary. $v_{2r2}^{\left(j\right)}$ is the second harmonic wave newly generated by the reflected $v_{1r}^{\left(j\right)}$. Therefore, the total reflected second harmonic, $v_{2r}^{\left(j\right)}$, will be obtained by adding $v_{2r1}^{\left(j\right)}$ and $v_{2r2}^{\left(j\right)}$. The reflected fundamental and second harmonic waves, $v_{1r}^{\left(j\right)}$ and $v_{2r}^{\left(j\right)}$, will be received by the receiver located at the center. The sample thickness is $z_{0}$. The solid-air interface can be regarded as the stress-free boundary, which provides the reflection coefficients of approximately $R_{1}=R_{2}=-1$ for the normally incidental fundamental and second harmonic waves. This assumption is based on the linear reflection of the nonlinear second harmonic wave and becomes a good approximation [5].

## 3. FZP Beam Fields and Definition of β

The Khokhlov–Zabolotskaya–Kuznetsov (KZK) equation or the Westervelt equation takes into account the combined effects of diffraction, absorption and nonlinearity, and is a proper model equation for sound beam radiation in fluids and their reflection from the stress-free boundary [20]. Recently, the quasilinear theory of the KZK equation and the Green’s function approach have been used to seek integral solutions for the reflected fundamental and second harmonic waves when a single element transducer was used [8,19,21]. The beam field formulation presented in this study is the straightforward extension of the previous methods used for a multiple element transducer. 

A KZK-type equation has been derived for longitudinal waves in isotropic solids [22] and used for calculating axisymmetrical sound beams in weakly nonlinear solid materials [1]. Here, we briefly present the quasilinear solutions for the KZK-type equation in closed forms. We finally provide the received average fields of the fundamental and second harmonic waves after being reflected from the boundary, from which the nonlinear parameter β can be defined for the pulse-echo mode.

We consider a longitudinal wave beam in an isotropic solid, and the nominal direction of propagation is along the *z* axis. The characteristic parameters of the beam are the annular piston source whose inner and outer radii are $a_{1}$ and $a_{2}$, respectively, the fundamental angular frequency ω, and the corresponding wave number k=ω/c, where c is the longitudinal wave velocity. 

Here, the KZK-type equation is used to describe longitudinal sound beams of finite amplitude in solids [22]. The KZK-type equation is similar to the KZK equation for nonlinear sound beams in thermoviscous fluids, and diffraction effects in both equations are taken into account within the parabolic approximation. Although the KZK-type equation is expressed in Lagrangian coordinates, whereas the corresponding KZK equation is expressed in Eulerian coordinates, this distinction is of a higher order than the approximations leading to these model equations, and it may therefore be ignored [22]. All analyses in this section are based on the KZK-type parabolic wave equation, which is the longitudinal counterpart of the KZK equation in nonlinear acoustics:

(1) $$\frac{\partial^{2}v}{\partial z\partial \tau }-\frac{c}{2}\nabla_{⊥}^{2}v-\frac{\delta }{2c_{}^{3}}\frac{\partial^{3}v}{\partial \tau^{3}}-\frac{\beta }{4c_{}^{2}}\frac{\partial^{2}v^{2}}{\partial \tau^{2}}=0$$

where v is the longitudinal particle velocity, τ=t−z/c is retarded time, $\nabla_{⊥}^{2}={\partial^{2}/\partial x}^{2}+{\partial^{2}/\partial y}^{2}$ signifies the transverse Laplacian and δ is an acoustic diffusivity [22]. Here, β is the nonlinearity parameter of the second harmonic in the isotropic solid. 

General integral expressions can be derived as the solutions to Equation (1) for the harmonic components due to weak finite amplitude radiation from axisymmetrical sources. Writing the total solution using the perturbation theory and assuming the time harmonic velocity yields the following quasilinear system of equations [1]:

(2) $$\frac{\partial V_{1}}{\partial z}+\frac{i}{2k}\nabla_{⊥}^{2}V_{1}+\alpha_{1}V_{1}=0$$

(3) $$\frac{\partial V_{2}}{\partial z}+\frac{i}{4k}\nabla_{⊥}^{2}V_{2}+\alpha_{2}V_{2}=\frac{\beta k}{4c}V_{1}^{2}$$

where $V_{n},\;n=1,2$ is the amplitude of the velocity of the *n*th harmonic and $\alpha_{n}=\delta n^{2}\omega^{2}/2c^{3}$ is the thermoviscous attenuation at frequency nω. 

### 3.1. Sound Beam Fields in the Forward Propagation Region

When the *j*th element of the FZP transmitter radiates the L-wave, the integral solutions of these equations can be constructed with Green’s functions:

(4) $$V_{1}^{\left(j\right)}(r,z)=2\pi \int_{0}^{\infty }V_{1}^{\left(j\right)}(r^{\prime },0)G_{1}(r,z|r^{\prime },0)\;r^{\prime }dr^{\prime }$$

(5) $$V_{2}^{\left(j\right)}(r,z)=\frac{\pi \beta k}{2c}\int_{0}^{z}\int_{0}^{\infty }{\left[V_{1}^{\left(j\right)}(r^{\prime },z^{\prime })\right]}^{2}G_{2}(r,z|r^{\prime },z^{\prime })\;r^{\prime }dr^{\prime }dz^{\prime }$$

where $G_{n}(r,z|r^{\prime },z^{\prime })$ is the Green’s function at frequency nω [21] and $r=\sqrt{{\left(x-x^{\prime }\right)}^{2}+{\left(y-y^{\prime }\right)}^{2}}$. Equation (4) can be solved for a known source function $V_{1}^{\left(j\right)}\left(r^{\prime },0\right)$. Once the fundamental solution $V_{1}^{\left(j\right)}\left(r,z\right)$ is obtained, the second harmonic solution $V_{2}^{\left(j\right)}\left(r,z\right)$ can be determined independently from Equation (5) since the source function for $V_{2}^{\left(j\right)}\left(r,z\right)$ is proportional to the volume distribution ${\left[V_{1}^{\left(j\right)}\left(r^{\prime },z^{\prime }\right)\right]}^{2}$. 

The source condition on the surface of the *j*th element is defined as:

(6) $$\{\begin{array}{l} V_{1}^{\left(j\right)}(r^{\prime },z^{\prime }=0)=V_{0}(r^{\prime },0),\;a_{1}^{\left(j\right)}\leq r^{\prime }\leq a_{2}^{\left(j\right)}\\ V_{2}^{\left(j\right)}(r^{\prime },z^{\prime }=0)=0 \end{array}$$

where $a_{1}^{\left(j\right)}$ and $a_{2}^{\left(j\right)}$ are the inner and outer radii of the *j*th annular element, respectively. In Equation (6), $V_{0}(r^{\prime },0)$ is a uniform distribution at the fundamental frequency *ω* and it is also assumed that the source does not radiate at the second harmonic frequency 2*ω*. 

The total amplitude of the propagated fundamental and second harmonic sound beams at a point (*r*, *z*) can be obtained by summing the contributions from all *N* elements:

(7) $$V_{n}(r,z)=\sum_{j=1}^{N}V_{n}^{\left(j\right)}(r,z),\;n=1,2$$

where n=1 and n=2 denote the fundamental and second harmonic waves, respectively.

### 3.2. Sound Beam Fields after Reflection from the Boundary

The expressions for the reflected velocity can be found by first calculating the fundamental and second harmonic velocities at the boundary ($z=z_{0}$), then multiplying by the appropriate reflection coefficients, and then integrating over the product of these new sources and the corresponding Green’s functions to sum the contributions from all source points. When the waves are reflected from a large planar interface, the stationary phase method [23] can be applied to simplify the expressions by using the initial velocity source. 

Then, the reflected velocity of the fundamental and second harmonics can be expressed as:

(8) $$V_{1r}^{\left(j\right)}(r,z)=R_{1}\left(2\pi \int_{0}^{\infty }V_{1}^{\left(j\right)}(r^{\prime },0)G_{1}(r,z|r^{\prime },0)\;r^{\prime }dr^{\prime }\right)$$

(9) $$\begin{array}{l} V_{2r}^{\left(j\right)}(r,z)=V_{2r1}^{\left(j\right)}(r,z)+V_{2r2}^{\left(j\right)}(r,z)\\ \;=R_{2}\left(2\pi \int_{0}^{\infty }V_{2}^{\left(j\right)}(r^{\prime },z_{0})G_{1}(r,z|r^{\prime },z_{0})\;r^{\prime }dr^{\prime }\right)+\frac{\pi \beta k}{2c}\int_{z_{0}}^{z}\int_{0}^{\infty }{\left[V_{1r}^{\left(j\right)}(r^{\prime },z^{\prime })\right]}^{2}G_{2}(r,z|r^{\prime },z^{\prime })\;r^{\prime }dr^{\prime }dz^{\prime } \end{array}$$

To calculate the received velocity at a distance *z* by a circular transducer of radius *b* which is concentric with the FZP elements, the concept of average velocity is used and calculated as follows:

(10) $${\tilde{V}}_{n}^{\left(j\right)}(z)=\frac{1}{\pi b^{2}}\int_{S_{R}}^{}V_{nr}^{\left(j\right)}(r,z)dS,\;n=1,2$$

where $V_{nr}^{\left(j\right)}(r,z)$ is computed by Equations (8) and (9).

The received fundamental and second harmonic amplitudes, Equation (10), resulting from the KZK-type equation represent the combined effects of the plane wave, diffraction and attenuation, and these effects cannot be easily separated. Therefore, we need to develop analytical expressions that provide explicit forms of these effects that can be used in a wide range of nonlinear acoustic applications, including sensor design. The multi-Gaussian beam (MGB) method [1,3,19] can be used to explicitly define these effects and Equation (10) can be expressed as:

(11) $${\tilde{V}}_{1}^{\left(j\right)}(z)=\left[V_{1}^{plane}(z)\right]\left[R_{1}M_{1}(\alpha_{1},z){\tilde{D}}_{1}^{\left(j\right)}(z)\right]$$

(12) $${\tilde{V}}_{2}^{\left(j\right)}(z)=\left[V_{2}^{plane}(z)\right]\left[R_{2}M_{21}(\alpha_{1},\alpha_{2},z){\tilde{D}}_{21}^{\left(j\right)}(z)+R_{1}^{2}M_{22}(\alpha_{1},\alpha_{2},z){\tilde{D}}_{22}^{\left(j\right)}(z)\right]$$

where the first term in each equation denotes the plane wave solution, $V_{1}^{plane}(z)=V_{0}$ and $V_{2}^{plane}(z)=\beta kV_{0}^{2}z/4c$, and the second term represents the combined effects of reflection, attenuation and diffraction. In Equations (11) and (12), $M_{1}$, $M_{21}$ and $M_{22}$ are referred to as the attenuation correction for waves $V_{1r}^{\left(j\right)}$, $V_{2r1}^{\left(j\right)}$ and $V_{2r2}^{\left(j\right)}$, respectively, and their closed-form expressions can be found in [19]. Here, $\alpha_{1}$ and $\alpha_{2}$ are the attenuation coefficients at the fundamental and second harmonic frequencies, respectively, at the source velocity $V_{0}$. Since the attenuation effects are derived from the plane wave solutions in an attenuating medium [1], it is noted that $M_{1}$, $M_{21}$ and $M_{22}$ do not depend on the specific element of the FZP transmitter. In Equations (11) and (12), ${\tilde{D}}_{1}^{\left(j\right)}$, ${\tilde{D}}_{21}^{\left(j\right)}$ and ${\tilde{D}}_{22}^{\left(j\right)}$ are referred to as the diffraction corrections for waves $V_{1r}^{\left(j\right)}$, $V_{2r1}^{\left(j\right)}$ and $V_{2r2}^{\left(j\right)}$, respectively, and their closed-form expressions can be found in [19].

The integral expression, Equation (8), which is the exact solution to the linear wave equation, represents the transducer radiation as a superposition of spherical waves radiating from point sources distributed on the plane $z^{\prime }=0$. Equation (9) also serves as an exact solution to the second harmonic wave equation in the quasi-linear theory of the KZK-type equation, since the exact linear solution is used in the right-hand side of Equation (9). Since Equations (8) and (9) are the multi-layer integral expressions for the fundamental and second harmonic waves, the calculation of these beam fields are computationally heavy, especially for the second harmonic wave. Equations (11) and (12) are numerically very efficient as they are the results of the MGB model approach, which represents integral solutions that are one layer less than the corresponding exact integration solutions. This is because in the MGB method, an integral is represented by the superposition of a small number (10–25) of Gaussian beams, whose properties can be described in analytical terms. 

Since the MGB method rests on the paraxial approximation, its accuracy was tested in the previous study [3]. The fundamental and second harmonic MGB solutions were obtained from the integral solutions for the quasilinear theory of the KZK equation and compared with the integral solutions for the quasilinear theory of the Westervelt equation, which is more exact than the parabolic KZK equation. The KZK equation is a nonlinear wave equation for finite amplitude sound beams in thermoviscous fluids. A finite size reception was considered when a single element transducer radiated a finite amplitude sound beam in water. The number of expansion coefficients for $A_{n}$ and $B_{n}$ used in the MGB method were 10 and 25, and their effects were also examined. Both of the fundamental and second harmonic fields calculated by the MGB method agreed well with the exact solution for most of the propagation distance used. The use of 25 expansion coefficients in the MGB calculations provided a better overall agreement. Based on these observations, the paraxial MGB method does not impose any restrictions in modeling the acoustic beam fields of the fundamental and second harmonic waves, providing accurate results as the exact solutions. Since both KZK and KZK-type equations are based on the same parabolic approximation, these observations also apply to the present study. In this study, 25 expansion coefficients were used in all simulation calculations.

### 3.3. Definition of Nonlinear Parameter

The total amplitude of the received fundamental and second harmonic sound beams at the initial source position ($z=2z_{0}$) can be determined by summing the contributions from all *N* elements:

(13) $${\tilde{V}}_{n}(z=2z_{0})=\sum_{j=1}^{N}{\tilde{V}}_{n}^{\left(j\right)}(2z_{0}),\;n=1,2$$

Now that the received velocity amplitudes of the fundamental and second harmonics are available, the nonlinear parameter β can be defined. Using the relationship between the particle velocity and the displacement, $\left|{\tilde{V}}_{n}\right|=n\omega {\tilde{U}}_{n},\;n=1,2$, the pulse-echo mode β at $z=2z_{0}$ can be expressed in terms of the received displacement amplitudes:

(14) $$\beta =\left[\frac{4{\tilde{U}}_{2}}{k^{2}z_{0}{\tilde{U}}_{1}^{2}}\right]\left[\frac{{\tilde{C}}_{T1}^{2}}{{\tilde{C}}_{T2}^{}}\right]=\left[\beta^{\prime }\right]\left[C_{T}\right]$$

where $\beta^{\prime }$ denotes the “uncorrected nonlinear parameter”, defined as the first term in the right hand side of Equation (14), and $C_{T}={\tilde{C}}_{T1}^{2}/{\tilde{C}}_{T2}$ is referred to as the “total correction”, which incorporates all the effects of reflection, attenuation and diffraction of the received fundamental and second harmonic waves. In Equation (14), $C_{T}$ is given by:

(15) $$C_{T}=\frac{\sum_{j=1}^{N}{\left[R_{1}M_{1}(2z_{0})\;{\tilde{D}}_{1}^{\left(j\right)}(2z_{0})\right]}^{2}}{\sum_{j=1}^{N}\left[R_{2}M_{21}(\alpha_{1},\alpha_{2},2z_{0}){\tilde{D}}_{21}^{\left(j\right)}(2z_{0})+R_{1}^{2}M_{22}(\alpha_{1},\alpha_{2},2z_{0}){\tilde{D}}_{22}^{\left(j\right)}(2z_{0})\right]}$$

## 4. Simulation of FZP Focused Beam Fields

The FZP consists of a set of transparent and opaque rings, and the waves diffracted by the transparent rings constructively interfere at the desired focus point. The radius of each ring element at a given frequency should be spaced using the following equation [14,15,16,17]:

(16) $$r_{n}=\sqrt{n\lambda \left(\frac{n\lambda }{4}+F\right)},\;n=1,\ldots ,N$$

where *r* is the radius of the ring, *n* is the order of the region, λ is the wavelength and *F* is the focal length. The radius of the center ring is $r_{1}$, the inner and outer radii of the second ring are $r_{1}$ and $r_{2}$, respectively, and so on. 

### 4.1. Effects of FZP Type

**Figure 2:**
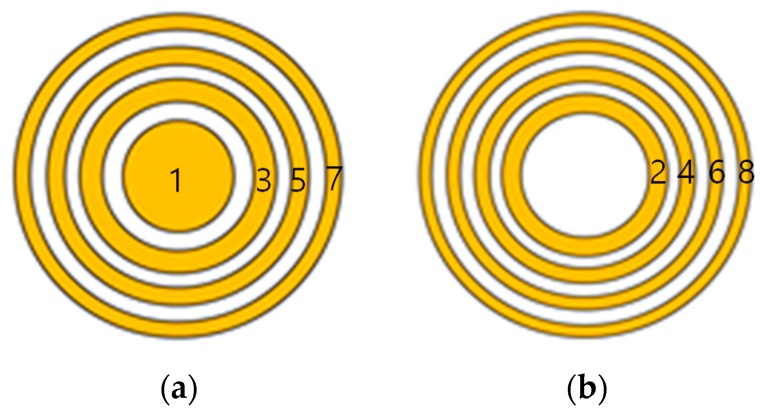
Two types of Fresnel zone plate: (**a**) Odd plate and (**b**) even plate.

There are two types of standard FZP, depending on the arrangement of elements used. The odd plate uses odd-numbered elements and the even plate uses even-numbered elements. Element number 1 denotes the center element. This number then increases sequentially when moving away from the center. Two types of standard zone plate are shown in Figure 2, where the yellow portion represents the transmitting zone and the white portion represents the blocking zone.

The propagation medium used in the simulation was aluminum 6061: The longitudinal velocity *c* = 6325 m/s, the density $\rho =2700\;{\mathrm{kg}/m}^{3}$ and the nonlinear parameter β=5.5. The fundamental frequency was *f* = 5 MHz and the source displacement was $U_{0}=1\times 10^{-9}$ m. We assumed a piston source excitation from each zone. The sample thickness used for optimal sensor design was $z_{0}=1\;\mathrm{cm}$. The attenuation effects on the calculated wave fields were not significant and have been ignored in all subsequent simulations.

In the first part of the simulation, we investigated the effects of FZP types on focusing performance. Two types of FZP were considered: Odd and even. Both FZPs have four concentric rings and were designed for a working frequency of 5 MHz and a focal length of 1 cm using Equation (16). The odd FZP consists of zones numbered 1, 3, 5 and 7, and the inner and outer diameters of each zone are calculated as $d_{1o}=7.2,\;d_{3i}=10.4,\;d_{3o}=12.9,\;d_{5i}=15.1,\;d_{5o}=17.1,\;d_{7i}=19,\;d_{7o}=20.8\;\mathrm{mm}$. The even FZP consists of zones numbered 2, 4, 6 and 8, and their dimensions are $d_{2i}=7.2,\;d_{2o}=10.4,\;d_{4i}=12.9,\;d_{4o}=15.1,\;d_{6i}=17.1,\;d_{6o}=19.0,\;d_{8i}=20.8,\;d_{8o}=22.5\;\mathrm{mm}$.

**Figure 3:**
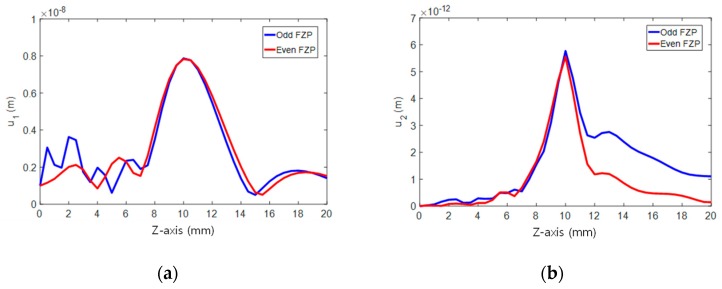
Comparison of focusing profiles between the odd FZP and the even FZP (*F* = 1 cm, *f* = 5 MHz, *n* = 4): (**a**) Fundamental wave and (**b**) second harmonic wave.

Waves radiate from each zone of the FZP transmitter and propagate in a half-space defined by z≥0. The on-axis distribution of the displacement was calculated using Equation (7) and the results are presented in Figure 3 with comparison between the odd- and even-type FZPs. The results for the fundamental wave are given in Figure 3a. The main focus occurs at the designed focal length in the two types of FZP. It can be said that there is little difference in focusing performance, except for the multiple secondary foci of lower amplitude appearing between the odd FZP surface and the main focus. 

The results for the second harmonic are given in Figure 3b, where both types show a narrower beam width and the main focus at the designed focal length. Compared to the even FZP, the odd FZP has a sidelobe appearing at a distance slightly greater than the primary focal length. In addition, the focused profile of the second harmonic tends to smooth much of the oscillatory sidelobes of the fundamental wave located at the front and back of the central main lobe. The focused wave properties are further analyzed below.

### 4.2. Effects of the Number of FZP Zones

In the second part of the simulation, we investigated the effects of FZP zone numbers on focusing performance. Two types of FZP were considered: Odd and even. The number of zones varied from 1 to 6 in each type of FZP. The fundamental frequency and the focal length were 5 MHz and 1 cm, respectively, the same as before. The dimensions of the elements constituting the FZP transmitter were obtained using Equation (16).

**Figure 4:**
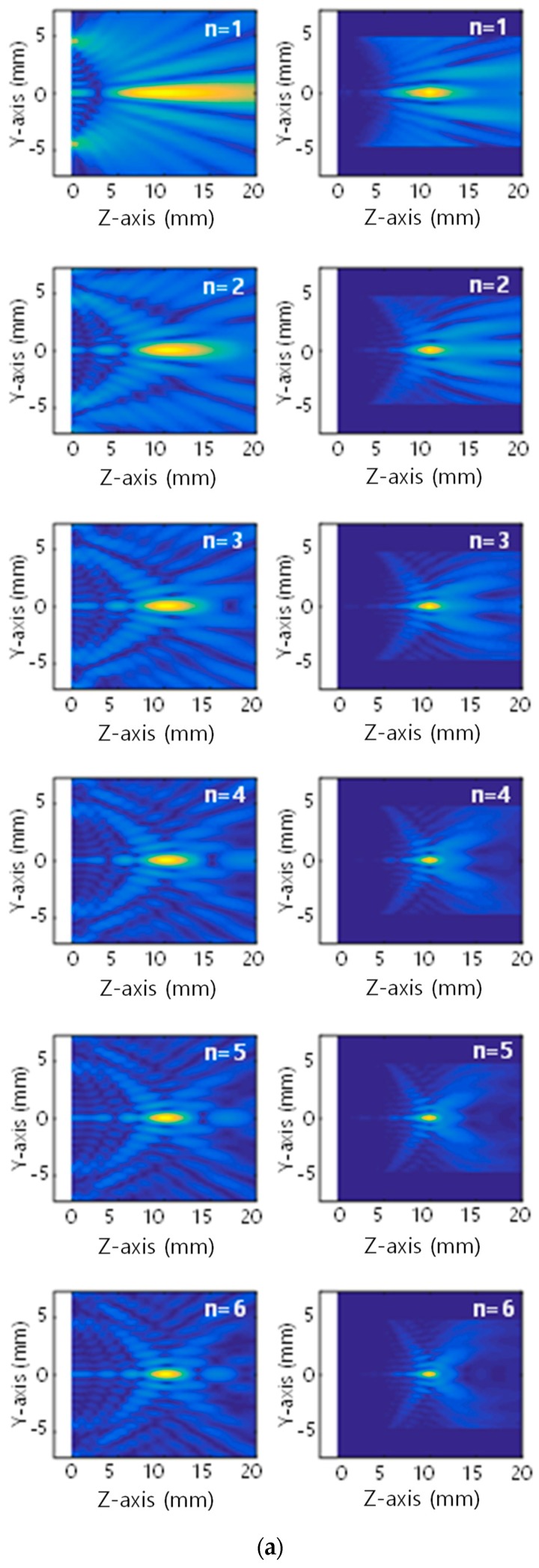

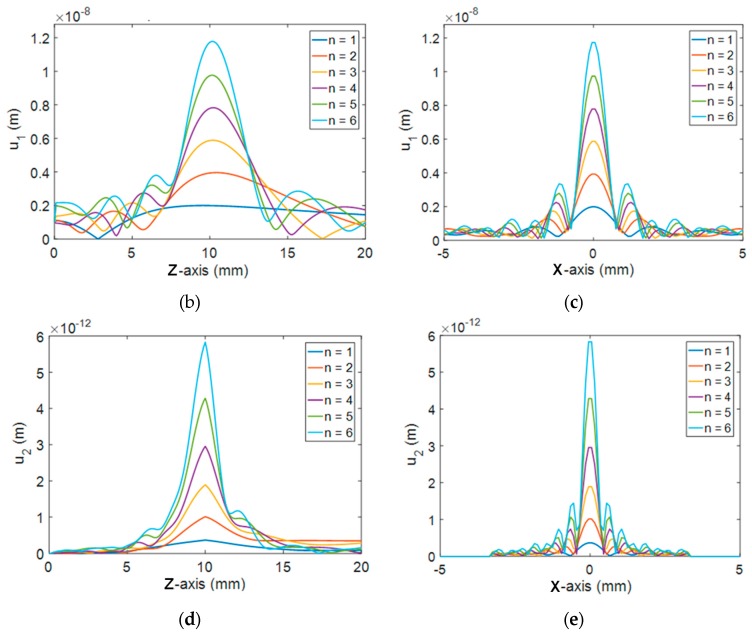
Simulated displacement distribution as a function of the number of Fresnel zones (*F* = 1 cm, *f* = 5 MHz, even FZP): (**a**) 2D beam profile of the fundamental (left column) and second harmonic (right column) waves, (**b**) on-axis distribution of the fundamental wave, (**c**) lateral distribution of the fundamental wave at the focal plane, (**d**) on-axis distribution of the second harmonic and (**e**) lateral distribution of the second harmonic at the focal plane.

Equation (7) was used as before for wave field calculation and the results are shown in Figure 4 for the even-type FZP as a function of the number of Fresnel zones. Figure 4a shows the 2D beam profile for the number of zones ranging from *n* = 1 to *n* = 6. Figure 4b,c shows the fundamental wave and the on-axis distribution and lateral distribution at the focal plane, respectively. Figure 4a shows that the diffracted wave focuses laterally, even with the use of a single zone, and the wave focuses more tightly at the designed focal length of *z* = 1 cm as the number of zones increases. The displacement amplitude at the main focus point increases linearly as the number of Fresnel zones increases, and accordingly the full width half maximum (FWHM) in both focusing profiles linearly decreases, as shown in Figure 4b–e. These properties are commonly observed in both fundamental and second harmonics.

The issue with the standard FZP is known as the relatively high noise floor or some constructive interference arising from the off-focus regions [16]. This is attributed to the fact that the FZP does not completely block diffracted waves that do not arrive in phase at the focus point. This trend is also present in the current simulation results, as multiple secondary foci of lower amplitude appeared between the zone plate and the main focus area, as shown in Figure 4b. The noise floor or the sidelobe grows as the number of zones increases, as shown in the lateral focusing profile (Figure 4c). 

Figure 4d,e shows the second harmonic and on-axis distribution and lateral distribution at the focal plane, respectively. Unlike the fundamental wave, the focused profile of the second harmonic tends to smooth most of the oscillatory sidelobes located at the front and rear of the central main lobe. As expected, the second harmonic profile has a narrower beam width in the on-axis and lateral distributions. 

The odd FZP shows similar behavior in the fundamental and second harmonic focusing profiles, but the results are not shown here due to space limitations. 

## 5. Optimal Design of FZP Sensors

### 5.1. Effects of Focal Length and Receiver Size

Because the acoustic parameters such as focal length and receiver size have great effects on the pulse-echo SHG, we first examined the effects of these parameters for 1 cm thick aluminum at a 5 MHz fundamental frequency using the four elements, with an odd-type FZP. Because a receiver is located at the center of the FZP sensors, all simulations below were performed using zone elements 3, 5, 7 and 9 of the odd-type FZP sensor. Elements 2, 4, 6 and 8 were used as before for simulations with the even-type FZP sensor. A total of six cases were considered in the simulation and are summarized in Table 1. The focal length was 1 cm for the first three cases and 2 cm for the next three cases. The receiver size in each group included an ideal point receiver (case 1 and 4), a 3 mm diameter receiver (cases 2 and 5) and a 6 mm diameter receiver (cases 3 and 6). The received second harmonic amplitude in each case was calculated using Equation (13).

**Figure 5:**
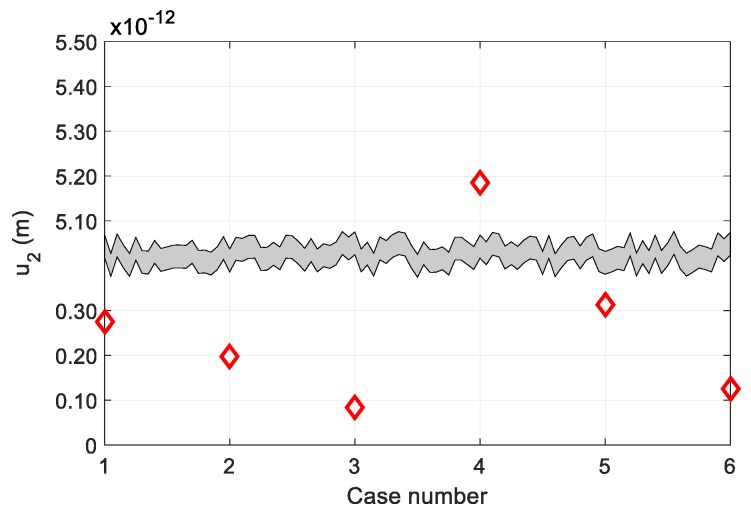
Dependence of pulse-echo SHG on focal length and receiver size.

Figure 5 shows the SHG simulation results for the six cases. The received second harmonic displacement in each group was greatest for the point reception case. Also, it was larger when the focal length was set at twice the sample thickness, i.e., at the initial transmission position. These results demonstrate that the second harmonic can vary greatly depending on the focal length and receiver size. Therefore, in order to efficiently generate and receive the second harmonic signal, an optimal sensor design should be performed using various combinations of acoustic parameters.

### 5.2. Optimal Design of FZP Sensors

The possibility of actually making the conceptual sensor is important and should be considered in conjunction with the optimal design. The fundamental frequency and receiver size were first selected as simulation parameters. As SHG is known to increase with increasing frequency, the fundamental frequency of 7.5 MHz was selected in addition to 5 MHz. The receive typically uses a broadband probe whose center frequency is twice the fundamental frequency. Considering the chosen fundamental frequencies, the possible receiver diameter was selected as 0.25 inch and 0.125 inch.

Next, we selected various focal lengths, ranging from 1 cm to 2 cm in 1 cm thick aluminum. The focal length of 1 cm corresponds to the reflecting boundary between the solid specimen and the air. 

Focal lengths of 1.3 cm, 1.5 cm and 1.7 cm correspond to the positions inside the specimen after reflection from the stress-free boundary. Finally, the focal length of 2 cm corresponds to the initial transmission position where the beam focuses after a round trip. Table 2 summarizes various cases of FZP sensors that can actually be fabricated with a combination of simulation parameters. Both odd- and even-type FZPs are considered in the simulations. Previous simulation results showed that the main focus properties of the FZP sensors are independent of the FZP type, so only one of the most likely types of optimal sensor was included in the even-type cases.

For a given frequency and focal length, the element size of the FZP transmitter was determined using Equation (16). Then, the received average displacements of the fundamental and second harmonics were calculated according to the receiver size using Equations (11) and (12). The total amplitudes of the received fundamental and second harmonics at the transmission position ($z=2z_{0}$) were calculated by adding the contributions from all four elements using Equation (13). Finally, the uncorrected nonlinear parameter, $\beta^{\prime }$, was calculated using Equation (14).

The optimal design criteria for the FZP sensor was determined by whether the second harmonic amplitude and the nonlinear parameter obtained in the pulse-echo (P-E) mode agreed well with the results of the corresponding through-transmission (T-T) method. In order to compare the P-E mode results with the T-T mode results, it is necessary to calculate the sound fields in the T-T mode, and the size of the transmitting and receiving transducers is required for this purpose. It can be assumed that the SHG process in the T-T mode is made by transmission and reception by a circular single element transducer of the same size. Then, the circular area of a T-T mode transducer is given as the whole area of the four element FZP transmitter. For convenience and simplicity of the problem, an odd-type FZP of 5 MHz frequency was assumed for all cases of simulation, where the diameter of the T-T mode transducer was calculated to be about 16.5 mm.

**Figure 6:**
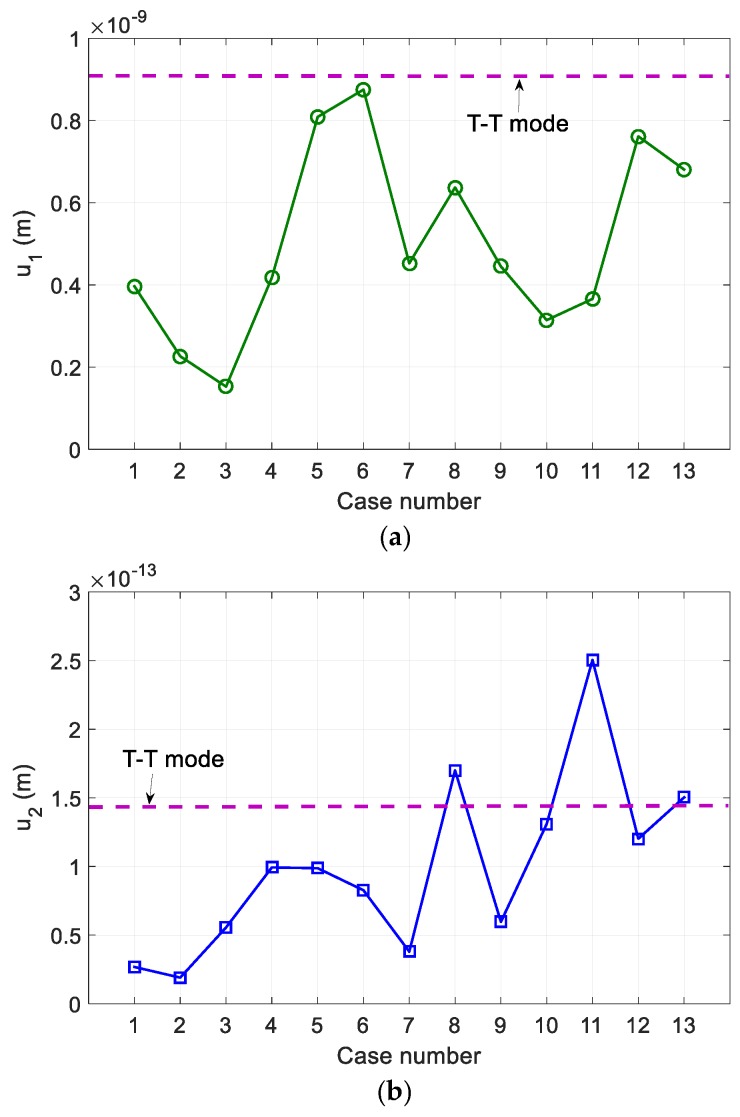
The received average displacement, calculated using the simulation parameters of Table 2: (**a**) Fundamental wave and (**b**) second harmonic wave. T-T mode denotes the through-transmission mode.

Figure 6 shows the simulation results for the fundamental and second harmonic displacement amplitudes according to the conditions of Table 2. All 13 cases considered provided a fundamental wave amplitude lower than the T-T mode, and only three of the considered cases provided a second harmonic amplitude greater than the T-T mode. These results show that the fundamental wave is the largest in case 6 and the second harmonic is the largest in case 11. For cases 6 and 11, the $\beta^{\prime }$ was calculated to be 1.75 and 30.35, respectively, which differs greatly from the reference β=5.5. This is due to the effect of the total correction, which indicates that the uncorrected nonlinear parameter is highly dependent on the total correction. Therefore, it is difficult to proceed with the optimal design only with the calculated second harmonic amplitude. When the FZP sensor is used to measure the second harmonic amplitude only, case 11 would be the best choice.

**Figure 7:**
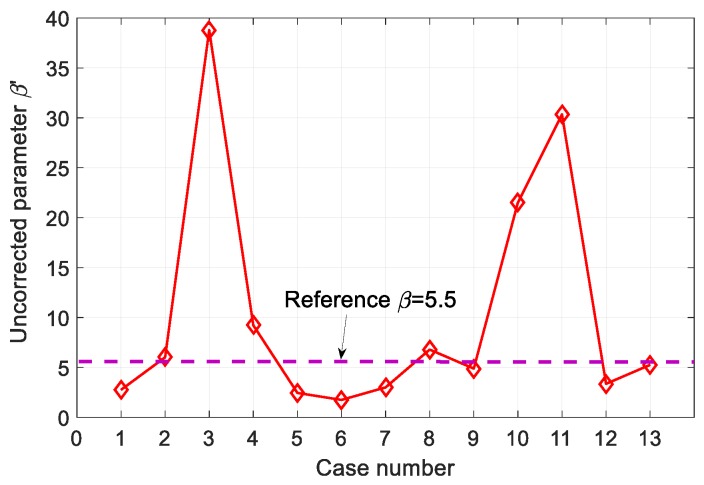
The uncorrected nonlinear parameter, $\beta^{\prime }$, calculated using the simulation parameters of Table 2.

In order to find the condition that the influence of total correction is small and the amplitude of the second harmonic has a magnitude similar to that of the T-T method, the uncorrected nonlinear parameter, $\beta^{\prime }$, was calculated for all conditions of Table 2 and the results are shown in Figure 7.

Based on the simulation results of Figure 6b and Figure 7, the second harmonic amplitude and uncorrected nonlinear parameter of cases 8 and 13 were found to be well matched to the T-T mode results. Thus, cases 8 and 13 provide the optimal design conditions for the P-E SHG measurements in 1 cm thick aluminum, i.e., the odd- or even-type FZP transmitter of four elements, at a 5 MHz fundamental frequency and a 2.0 cm focal length, using a 0.125-inch diameter receiver. If improved second harmonic generation is an important goal for the design of a given sensor, case 11 is best suited for this purpose and can generate a second harmonic amplitude that is approximately 1.7 times larger than the T-T mode.

### 5.3. Effects of Sound Velocity Change

It is assumed that the sound velocity of a solid medium is constant in Equation (16). However, due to the possibility of heterogeneity of the actual material, the actual velocity may vary slightly from the velocity used in the sensor design. Therefore, it is necessary to investigate the effect of sound velocity change on the second harmonic amplitude and the uncorrected nonlinear parameter. The velocity variation of the medium was considered to be 100 m/s smaller than 6325 m/s and 100 m/s larger than 6325 m/s. The change is about 1.6%. The effects of each variation were simulated using the determined optimal design conditions of cases 8 and 13.

The nonlinear parameter is defined as $\beta =3+C_{111}/\rho c_{}^{2}$, where $C_{111}$ is the third order elastic constant, ρ is the density and c is the longitudinal wave velocity of the solid specimen. Assuming that ρ and $C_{111}$ are constants, then a ∓100 m/s variation in the sound velocity will change the β values to 5.58 and 5.42, respectively. This is about a 1.45% change in β. These new β values and changed sound velocities were utilized in the simulation, using the FZP sensor designs of cases 8 and 13, leaving the other parameters unchanged.

**Figure 8:**
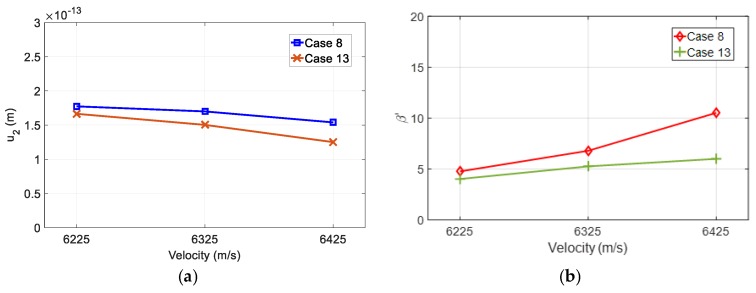
Effects of ∓100 m/s velocity change of the propagation medium on: (**a**) The second harmonic amplitude and (**b**) the uncorrected nonlinear parameter.

The second harmonic amplitude and the resulting uncorrected nonlinear parameter due to the velocity change of the propagation medium were calculated and the results are shown in Figure 8. Figure 8a shows that the actual velocity is less than the design velocity, slightly increasing the amplitude of the second harmonic. An actual velocity larger than the design velocity reduces the amplitude of the second harmonic slightly more than this, and this change is more severe in even-type FZP sensors. However, in the case of $\beta^{\prime }$ shown in Figure 8b, the actual velocity, when it is lower than the design velocity, slightly reduces $\beta^{\prime }$, but an actual velocity higher than the design velocity increases $\beta^{\prime }$ even further, and this change is very serious in case of odd-type FZP sensors.

These simulation results indicate that it is important to use a velocity value close to the actual velocity of the test specimen in the FZP sensor design for P-E SHG measurements. The velocity change of the specimen changes the focusing characteristics of the designed FZP sensor, and a FZP sensor with the fixed focal length can hardly actively cope with this velocity change. The fixed focal length is a disadvantage of the FZP sensor and needs to be overcome in the future.

In the optimal design of FZP sensors, both odd- and even-type FZP transmitters exhibit similar behavior in P-E SHG measurements, such that both designs can be used without much difference if there are no constraints other than the basic design requirements. However, even-type FZP designs may be preferred for P-E SHG measurements because such designs are less sensitive to changes in the sound velocity of specimens.

## 6. Conclusions

This paper explored the optimal sensor design for pulse-echo SHG and *β* measurements using Fresnel zone plate (FZP) focused beams. The conceptual design of a sensor configuration uses separate transmission and reception methods, where a broadband receiver is located at the center and a four-element FZP transmitter is positioned outside the receiver to create a focused beam at the specified position in a solid sample. Comprehensive simulations have been performed for focused beam field analysis and an optimal sensor design was found using various combinations of focal length, receiver size and frequency. It was shown that the optimally designed sensors for 1 cm thick aluminum could produce the desired second harmonic amplitude and the resulting $\beta^{\prime }$ value corresponding to the through-transmission method. This optimum design can be applied without a loss of accuracy, even if the actual velocity of the specimen is slightly different from the designed value. If improved second-harmonic generation is most important factor in the design of a given sensor, other designs can be used to produce a second harmonic amplitude that is approximately 1.7 times greater than what the transmission method provides.

The odd- and even-type FZP sensors provided similar behavior in the main focus properties, and their optimal designs also showed similar behavior in the pulse-echo SHG measurements. Thus, both optimal designs can be used without much difference if there are no constraints other than the basic design requirements. However, even-type FZP designs may be preferred in the case of pulse-echo SHG measurements, because such designs have smaller external dimensions and are less sensitive to changes in the sound velocity of specimens.

The designed FZP transmitter in this study consisted of four concentric rings and could be interconnected to operate as a single focusing element, unlike traditional phased array transducers, making it more economical to build the experimental devices. All the results presented in this paper are based on simulations and it is necessary to fabricate the probe and carry out experimental verification. The production of the optimum design transducer is underway.

## Figures and Tables

**Table 1:** Various combinations of focal length and receiver type in FZP sensors.

Case No.	Focal Length (mm)	Receiver Type
1	10	Point
2		Area (diameter of 3 mm)
3		Area (diameter of 6 mm)
4	20	Point
5		Area (diameter of 3 mm)
6		Area (diameter of 6 mm)

**Table 2:** Various combinations of focal length, receiver size and frequency for optimal design of FZP sensors.

Case No.	FZP Type	Focal Length (cm)	Receiver Diameter (inch)	Frequency (MHz)
1	Odd	1.5	0.25	5
2	Odd	1.7	0.25	5
3	Odd	2.0	0.25	5
4	Odd	1.0	0.125	5
5	Odd	1.3	0.125	5
6	Odd	1.5	0.125	5
7	Odd	1.7	0.125	5
8	Odd	2.0	0.125	5
9	Odd	1.3	0.125	7.5
10	Odd	1.5	0.125	7.5
11	Odd	1.7	0.125	7.5
12	Odd	2.0	0.125	7.5
13	Even	2.0	0.125	5
